# Coexistence of Granular Cell Tumor with Squamous Cell Carcinoma on the Tongue: A Case Report 

**Published:** 2015-01

**Authors:** Recep Bedir, Rukiye Yilmaz, Ibrahim Sehitoglu, Abdulkadir Ozgur

**Affiliations:** 1 *Department of Pathology, Recep Tayyip Erdogan University of Medical Faculty, Rize, Turkey.*; 2 *Department of Otorhinolaryngology,Recep Tayyip Erdogan University of Medical Faculty, Rize, Turkey.*

**Keywords:** Immunohistochemistry, Granular cell tumor, Squamous cell carcinoma, Tongue.

## Abstract

**Introduction:**

Granular cell tumors (GCTs) are rare and mostly benign soft tissue tumors. Though they have been reported in all parts of body, they are generally located in the head and neck region, especially on the tongue. Some malign forms exist, but these have been rarely reported. Granular cell tumors have a neural origin and, in immunohistochemical evaluations, they express S-100 and neuron specific enolase (NSE). The treatment of these tumors is bulky surgical excision.

**Case Report:**

In this case, a cauliflower shaped lesion with a 1 cm diameter was excised from the midline tongue of a 65 year old woman. The histopathological evaluation indicated that it was squamous cell carcinoma (SCC) covering GCT. Herein, the coexistence of GCT and SCC we describe on the same region of the tongue, in accordance with literature review, since this is a very rare condition.

**Conclusion:**

Pseudoepitheliomatous hyperplasia may accompany GCTs on the tongue and this condition may mimic well-differentiated SCC. For this reason, with the help of Ki-67 and p63 expression, in addition to immunohistochemical markers, well-differentiated SCC should be differentiated from pseudoepitheliomatous hyperplasia through careful investigation.

## Introduction

Granular cell tumors (GCTs) were first described by Abrikossoff in 1926 (1). Granular cell tumors are rare soft tissue tumors, which are usually benign. Though they have been reported throughout the body, they are generally located in the head-neck region and especially on the tongue (2). Interestingly, GCTs may coexist with other malign neoplasms. Herein, the coexistence of squamous cell carcinoma with a simultaneously diagnosed granular cell tumor, located on the same region(the tongue) we present in accordance with literature review, especially regarding the 3^rd^ case in literature. 

## Case Report

**Fig 1a:**
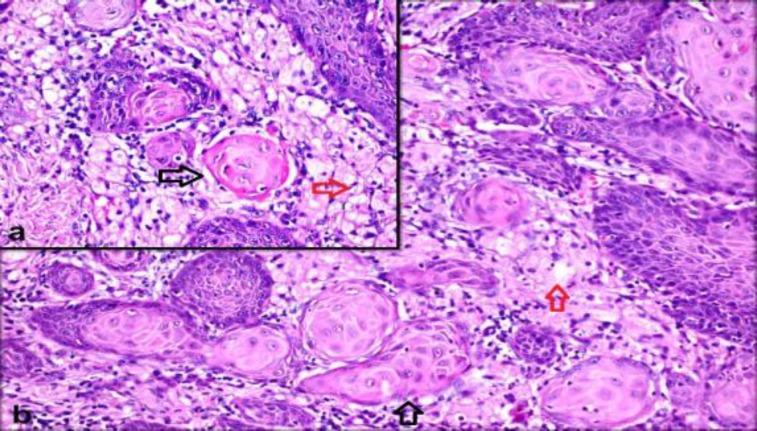
Squamous islands consisting of atypical squamous cells with large hyperchromatic nucleus, marked nucleolus and mitosis, invading superficial dermis (black arrow) (H&E,x200) **Fig1b:**GCT consisting of cells with large granular cytoplasm and oval-round nucleus located (red arrow) under the squamous cell carcinoma on dermis (H&E, x400)

A sixty-five year old female patient was admitted to the outpatient clinic with a lesion on her tongue, which had been present for 1 year but had enlarged in the last 3-4 months. During her physical examination, a cauliflower shaped mass with a 1 cm diameter was observed in the midline of the tongue. Due to suspected malignancy, large total excision was performed on the mass. During microscopic evaluation, well-differentiated squamous cell carcinoma was diagnosed, which was superficially acantho- tic, hyperkeratotic, and papilloma- tosis. In addition, it was invading the dermis in some places and consisted of atypical squamous cells in a few areas where it was invading the dermis as free squamous islands (Fig1.a). 

 Inside the connective tissue under the squamous cell carcinoma, a different infiltration consisting of polygonal cells with oval-spherical small nuclei and large granular cytoplasms, forming solid layers was observed (Fig1.b). 

**Fig 2:**
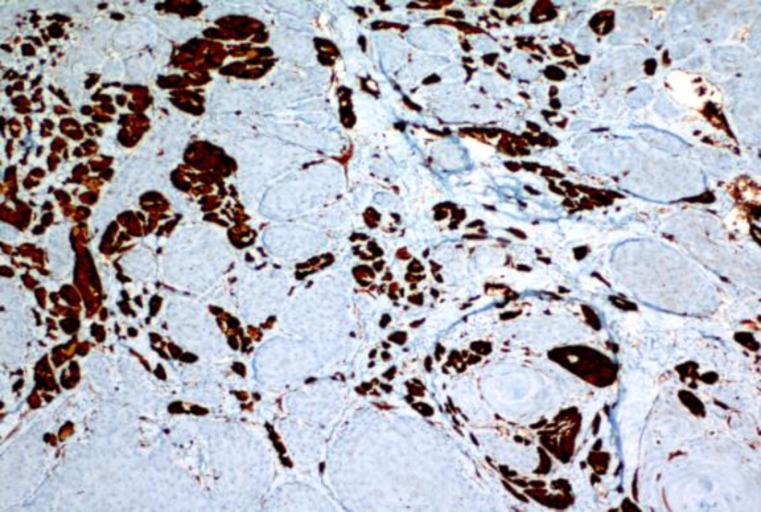
GCT was observed strongly positive with S-100 (x100)

 During immunohistochemical evaluation of these cells, it was observed that they were diffuse and strongly positive with S-100 (Fig. 2) and neuron-specific enolase (NSE), but only faintly positive with CD68. 

**Fig 3:**
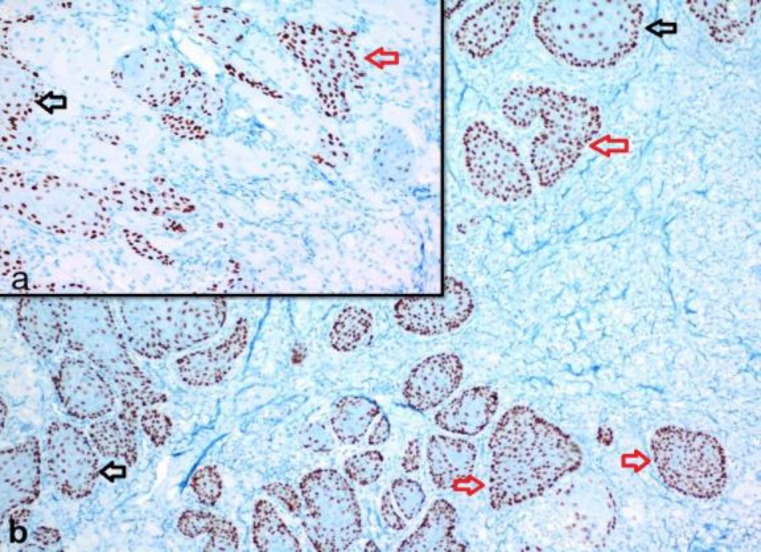
While a diffuse and full-layered positive staining with Ki-67 is present on epithelium of atypical squamous islands (red arrow), a limited staining is present on basal-parabasal layers of non-neoplastic epithelium (black arrow) (Ki-67, x200)

During histochemical evaluation, the cytoplasmic granules of these cells were positive for periodic acid-Schiff (PAS) and resistant to diastase. The Ki-67 proliferation index was 1% in GCT, although nuclear positive staining was observed on all epithelial thickness of in situ and invasive foci of squamous cell carcinoma (Fig. 3).

**Fig 4:**
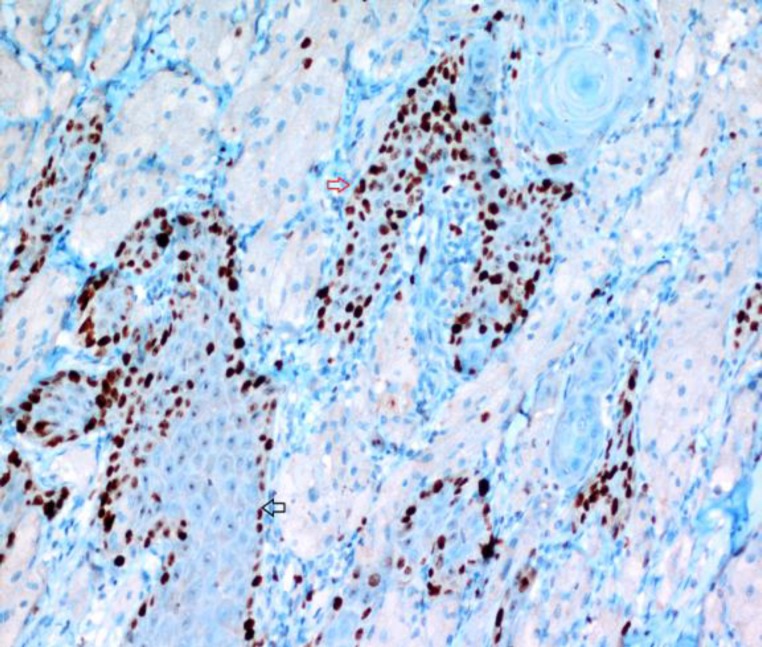
While a diffuse and strong positive staining with p63 is present on atypical squamous islands (red arrow), a limited staining is present on basal-parabasal layers of non-neoplastic epithelium (black arrow) (p63, x200)

However, a limited Ki-67 staining was observed only on the basal-parabasal layers of the pseudoepitheliomatous hyperplasia foci around the squamous cell carcinoma. During immunohistochemical evaluation of squamous cell carcinoma using P63, a similar staining pattern was observed with Ki-67 (Fig.4). 

In GCT neither pleomorphism, nor mitosis, nor increase in cellularity or necrosis were determined. According to both morphological and immunohistochemical findings, the case was diagnosed as squamous cell carcinoma developing on a granular cell tumor. Cervical lymph node was not detected by physical examination and ultrasonographic examination. 

The chest X-ray was reported as normal. The patient underwent operation in the clinical stage of T1N0M0. The tumor close to the surgical margins was receding. Surgical intervention was performed under general anesthesia. 

**Fig 5:**
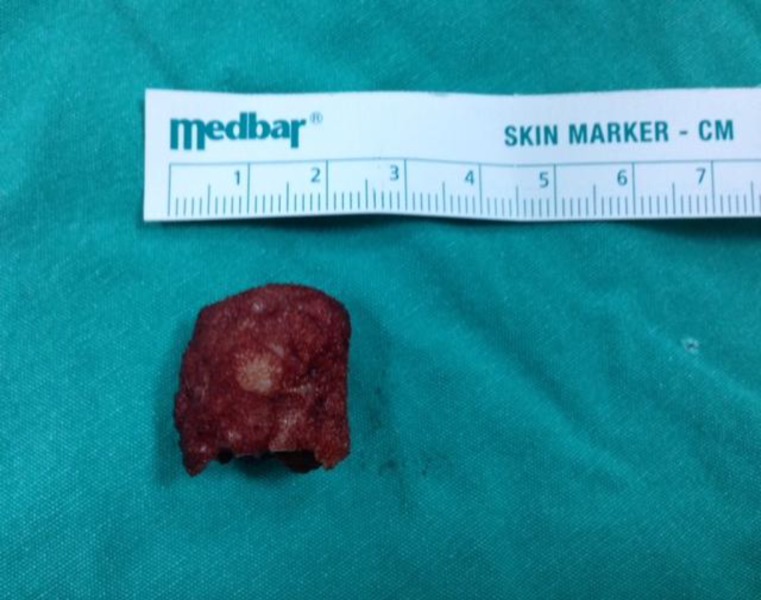
Re-excised mass in a diamond shape

The tumor was re-excised; leaving a surgical safety margin of 1 cm from all directions to the previous surgical area (Fig.5). After a 3-month follow-up of the patient, no recurrence was observed.

## Discussion

Granular cell tumors are generally asymptomatic and commonly reported in the 4-6^th^ decades of life. First described by Abrikossoff, they were qualified as tumors originating from muscle and were named as granular cell myoblastoma. Later, other investigators reported that they have histiocytic, fibroblastic, myoepithelial, and neuronal origins. Recently, the data of S-100 and NSE expression of these tumors support us to judge these tumors as benign neural cell tumors. The granular appearance of tumor cells depends on the high number of lysosomes in the cytoplasm. However, the tumor hystogenesis is still not obviously known. Though GCTs are usually reported in the head-neck region, they can be seen in many other parts of the body including the skin, subcutaneous tissue, the larynx, the trachea, the bladder, the uterus, the vulva, and the central nervous system. Nevertheless, approximately 30-60% of the cases are reported on the tongue (1-3). Typically they are small, solitary lesions, with a diameter rarely larger than 3 cm. In literature, malignant types of GCTs have also been reported, but they are extremely rare and constitute 1.2% of all GCTs. Malignant lesions typically metastasize to the lung, liver, and bone through lymphatic and hematogenous ways (2).There is an interesting association between GCTs and other malign neoplasms. In literature, GCTs and some tumors have been defined on the same organ together. Saito et al. and Vinco et al. reported esophageal GCT together with squamous cell carcinoma (SCC). Al-Ahmadie et al. on ipsilateral breast, and Tai et al. on contralateral breast, reported the coexistence of GCT with invasive ductal carcinoma (5-8). Similar to this case Caltabione et al. and Son et al. have reported the coexistence of GCT with SCC on the same region: the tongue(3,9). Other neoplasms reported together with the GCTs in literature are summarized on (Table.1). 

**Table 1:** Brief summary of other malignancies reported in the literature in association with GCT

**References**	**Age**	**Sex**	**Immunohistochemical findings**	**Treatment**	**Localization**	**Associated in** **tumor type**	**Follow-up**
Saito et al.^5^	69	M	GCT:S-100(+),NSE(+), Vimentin(+),SMA(-), Desmin (-), P53 (-) SCC: EMA (+), CK(+) Ki-67 (+), p53 (-)	partial esophagectomy with two-field (thoraco-abdominal) lymph node dissection	esophagus	intramucosal squamous cell carcinoma with minimal invasion	NS
Vinco et al.^6^	44	M	GCT: S-100 (+)	subtotal esophagectomy with gastroplasty	esophagus	squamous cell carcinoma	NS
Al-Ahmadie H et al.^7^	57	F	invasive ductal carcinoma: estrogen receptor (+) progesterone receptor (-), and HER-2/neu protein(-),CK (+) GCT: S-100 (+), CK (-).	excisional biopsy with a right axillary nodal dissection,	right breast	invasive ductal carcinoma	9 months
Caltabiano et al. ^9^	47	M	SCC:p53 and P63 strongly (+) GCT: S-100 (+)	excisional biopsy with nodule dissection	tongue	squamous cell carcinoma	12 months
Sony et al.^3^	27	F	SCC: CK(+), Ki-67 and P53 strongly (+) GCT:S-100(+), vimentin (+), NSE (+), SMA (-),CK (-)	Excisional biopsy	tongue	squamous cell carcinoma	12 months
Current case	55	F	SCC: CK(+), Ki-67 and P63 strongly (+) GCT:S-100(+),NSE(+), SMA (-), CK (-)	Excisional biopsy	tongue	squamous cell carcinoma	3 months

To the best available knowledge, this is the 3^rd^ case reported in English literature, which demonstrates the coexistence of GCT with SCC localized on the same region, on the midline of the tongue. Pseudoepit- heliomatous hyperplasia or pseudo- carcinomatous hyperplasia generally accompany the overlying mucosa of GCTs. (7,9) Therefore, the differential diagnosis of well-differentiated squamous cell carcinoma should be preformed especially carefully. In squamous cell carcinoma, atypical squamous islands with marked atypia, anaplasia, and mitotic activity are observed in the dermis. Sometimes this differentiation is not easy and occasionally immuno- histochemical evaluations may be necessary. Caltabione et al. (9) proposed that the immunohistochemical marker p63 might be beneficial in the differentiation of SCC with pseudoepitheliomatous hyperplasia. In this case report, since pseudoepit- heliomatous hyperplasia was observed, p63 was used together with Ki-67, for differential diagnosis. While diffuse and strong staining was observed on invasive foci with both markers, only limited staining on parabasal-basal layers was detected in non-neoplastic regions. In GCT, the Ki-67 proliferation index was determined as low (1%). The differential diagnosis of GCT with verrucous xanthoma, a rare lesion of the oral mucosa, is performed with the S-100 and NSE expression.

In literature, it has been reported that GCT pathogenesis is associated with alcohol abuse, which was present in some case histories. As evidence of this, it has been suggested that ethanol stimulates myelin formation and Schwann cell proliferation in vitro. At the same time alcohol plays a role in SCC pathogenesis. Some researchers speculate that the pathogenesis of the overlying type may involve chronic irritation of the mucosa, leading to carcinoma development. On the other hand, the association of GCT with other malign tumors cannot be elucidated yet (3,6,9). This case had a history of smoking half a pack per day for about 30 years; but there was no alcohol abuse history. 

In treatment of GCTs, conservative surgical excision is recommended. A smaller than 1 cm surgical margin may be necessary on benign GCTs, while the surgical margin should be higher than 2 cm on malignant ones or on ones that are suspected to be malignant. On benign GCTs, no more additional treatment or follow-up of patient is necessary after surgical excision. However, recurrent, rapidly growing tumors, and tumors with a diameter larger than 5 cm should be followed due to suspected malignancy (3,4,6,9) In this case, the tumor was terminating close to the surgical margins. For this reason, the mass was re-excised in a diamond shape.

## Conclusion

Pseudoepitheliomatous hyperplasia may accompany the surfaces of GCTs on the tongue and this condition may mimic well-differentiated SCC. For this reason, with the help of Ki-67 and p63 expression, in addition to immunohistochemical markers, well-differentiated SCC should be differentiated from pseudoepitheliomatous hyperplasia through careful evaluation. 

## Key Messages

Pseudoepitheliomatous hyperplasia or pseudocarcinomatous hyperplasia generally accompany the overlying mucosa of GCTs. Therefore, the differential diagnosis of well-differentiated squamous cell carcinoma must be performed especially carefully.
